# Rapid DNA visual detection of polymicrobial bloodstream infection using filter paper

**DOI:** 10.1038/s41598-022-08487-4

**Published:** 2022-03-16

**Authors:** Yajing Song, Peter Gyarmati

**Affiliations:** Department of Cancer Biology and Pharmacology, University of Illinois College of Medicine, Peoria, IL 61605 USA

**Keywords:** Biochemistry, Biotechnology, Microbiology, Molecular biology, Molecular medicine, Materials science, Nanoscience and technology

## Abstract

Bloodstream infection (BSI) is a major complication in patients with cancers due to therapy-induced neutropenia and underlying conditions, which increases hospitalization time and mortality rate. Targeted and timely antimicrobial management is crucial to save the patients’ lives and reduce the social and economic burdens. Blood culture is a routine clinical diagnostic method of BSI with a long turnaround time, and generally identifies monomicrobial BSI. Thus, polymicrobial BSI often goes undetected although it occurs more frequently in these patients and results in more severe outcomes compared to monomicrobial BSI. In this work, we apply glutaric anhydride, *N*-hydroxysuccinimide and *N,N′*-dicyclohexylcarbodiimide to fabricate a functional surface on cellulose filter paper. Targeting three pathogens (*Escherichia coli*, *Saccharomyces cerevisiae*, and human cytomegalovirus) commonly occurring in BSI in neutropenic patients, we demonstrate rapid and accurate triplex pathogen DNA detection using the functionalized paper. All three pathogen DNA was identified in 1–5 min with a detection limit of 0.1–0.5 ng/µL. The developed test tool has the potential to provide rapid polymicrobial BSI diagnosis in support of timely, accurate antimicrobial treatment, and could be integrated into an automatic sample-to-result portable equipment.

## Introduction

Bloodstream infection (BSI) is an infection defined by the presence of viable microorganisms in the blood, and it poses a significant burden on the healthcare of the United States with around 580,000–680,000 cases per year^1^. The immune system in physiological conditions is able to quickly fight against pathogens in the bloodstream via innate immunity and proinflammatory cytokines to avoid sepsis occurrence^2,3^. However, patients with cancers usually experience neutropenia either due to anticancer treatment such as chemotherapy, underlying medical conditions such as hematologic malignancies, or both. Neutropenia increases patients’ risk of BSI progressing to sepsis, especially in polymicrobial BSI. Polymicrobial BSI primarily results from bacteria, occasionally from fungi and/or viruses, and contributes more severe outcomes compared to monomicrobial BSI^4–10^. Rapid and appropriate antimicrobial therapy saves more patients’ lives than any other clinical intervention^8–12^ as long as it’s initiated within 1 to 3 h of symptom-onset from suspected BSI. Therefore, effective identification of causative pathogens is of primary importance to ensure timely and targeted antimicrobial therapy^13^.

The current clinical standard approach for BSI diagnosis—blood culture—takes hours to days to complete and needs large sample volumes (5–30 mL), yet primarily detects monomicrobial infection^14,15^. Compared to culture-dependent methods, nucleic acid amplification tests (NAATs) are more rapid and sensitive, and the volume of sample that NAATs require is less than 1 mL. Moreover, NAATs can simultaneously identify single or multiple pathogens, which are more efficient than culture for the patients who have been treated with antibiotics^16–20^. SeptiFast, SepsiTest, VYOO, and PLEX-ID are the most common NAAT commercial systems for BSI diagnosis. However, these assays are costly. For example, the cost of SeptiFast is approximately $300/assay; the equipment of PLEX-ID is around $200,000; and all systems require extensive instrumentation and trained staff^20–22^. To simplify the process of BSI diagnosis and reduce cost, we have developed a lateral flow test tool utilizing specific three-dimensional structural and natural properties of cellulose filter paper for rapid and accurate singleplex DNA detection, which is cost-efficient and easy to operate^23^. In this study, we report a rapid and accurate multiplex DNA (bacterium, fungus, and virus) detection using functionalized cellulose filter paper, which can rapidly provide information on multiple pathogens to support appropriate BSI antimicrobial administration.

## Materials and methods

### Materials and reagents

Genomic DNA (gDNA) of *E. coli*, *S. cerevisiae*, human serum, DNA oligonucleotides (Tables S1–S3), and chemicals including glutaric anhydride (GA), *N*-hydroxysuccinimide (NHS), and *N,N′*-dicyclohexylcarbodiimide (DCC), dimethyl sulfoxide (DMSO), *N,N*-dimethylformamide (DMF), SSC buffer, and SDS were purchased from Sigma-Aldrich. HCMV gDNA was ordered from ATCC. Whatman™ qualitative filter paper, DYNAL MyOne Dynabeads Streptavidin C1, 2× PCR SYBR Green master mix, myImageAnalysis software, and Qubit 3.0 fluorometer were purchased from Fisher Scientific. A Qiagen Blood DNA extraction kit and Rotor-Gene 6000 instrument were used. A custom-made magnetic stand (MagRach 16), 2× Kapa PCR readymix (Kapabiosystem), and Techne primer thermal cycler (Techne) were used.

### Methods

#### Functionalization of cellulose filter paper

We have previously developed a surface chemistry to activate filter paper to immobilize oligonucleotide DNA^23^. Briefly, by reacting to 1.75 M of GA in DMF overnight at room temperature, the hydroxyl groups on cellulose surface transformed into carboxylic groups. After washing with DMF and deionized water, reacting 500 nM of NHS and DCC in DMSO for 4 h at room temperature further transformed the carboxylic groups into ester groups on the surface. After washing with DMSO and deionized water, the formed functional groups on cellulose surface were fixed with methanol overnight. The steps of carboxylation with 1.75 M of GA in DMF and esterification with 500 nM of NHS and DCC in DMSO were repeated on the same paper slide to increase the final concentration of functional groups.

#### Design of primers and probes

The *Escherichia coli* (*E. coli)* O157:H7 primers were designed with primer-BLAST^24^ based on its specific region by aligning to multiple genomes of *E. coli* O157:H7, *E. coli* K12, *E. coli* APEC O1, *Enterobacter* 638, *Salmonella* typhi, *Salmonella enterica* paratype ATCC 9150, *Shigella flexneri* 2a, *Yersinia pestis* CO92, *Blochmannia floridanus*, and *Buchner* sp. in the UCSC genome browser^25^. The forward primer^26^ and reverse primer of Human cytomegalovirus (HCMV) were designed based on a highly conserved gene: the major immediate-early (MIE) gene. Fungal primers of ITS3 (forward) and ITS4 (reverse) were used to amplify the internal transcribed spacer (ITS) region of *Saccharomyces cerevisiae* (*S. cerevisiae*)^27^. Biotin modification was added to the 5′ ends of the forward primers (Table S1). Three specific detection probes were designed within the amplicons of *E. coli*, *S. cerevisiae*, and HCMV, excluding primer sequences (Table S2). APT_2_, a synthetic sequence^23^, was a negative control probe (NTC).

#### Immobilization of probe oligonucleotides

Four probes were with amino-modifier C12 at 5′ ends (Table S2)^23^ were printed manually targeting *S. cerevisiae*, HCMV, *E. coli*, and APT_2_ on each functionalized paper slide (15 mm × 20 mm). Specifically, the functionalized cellulose filter paper slide was successively printed using 3 µL of each 20 µM of probe in 1× printing buffer (0.05 mM sodium phosphate, pH 8.5) manually, incubated in a humid chamber overnight, blocked using 50 mM ethanolamine with 100 mM Tris (pH 9.0) at 55 °C for 30 min, washed with 4 × SSC containing 0.1% SDS at 55 °C for 30 min, dried, and processed for detection^23^.

#### Optimization of primer concentrations and PCR annealing temperature

PCR reactions in 1× Kapa PCR readymix were used to prepare the amplicons on a Techne primer thermal cycler. Four different concentrations of each pair of primers (100 nM, 200 nM, 300 nM and 400 nM) and four different annealing temperature (50 °C, 53 °C, 57 °C, and 60 °C) were assessed to optimize concentration of each pair of primers for each pathogen and to confirm one optimal universal annealing temperature for the amplification of all pathogens. Briefly, by comparing the *S. cerevisiae* amplicon from the different primer pair concentrations at the same annealing temperature, we confirmed the optimal concentration of *S. cerevisiae* primers. We then narrowed annealing temperature to two by comparing the four different ones with the confirmed concentration of *S. cerevisiae* primers. Based on the best two annealing temperature of *S. cerevisiae*, we confirmed the final annealing temperature and the concentrations of HCMV and *E. coli* primers, respectively.

#### Construction of standard curves for real-time PCR

Standard curves were constructed in 25 µL reaction volume in a real-time quantitative PCR assay on a Rotor-Gene 6000 instrument. The reaction mixture contained 1× PCR SYBR Green master mix, the optimal concentration of each pair of primers (200 nM of *E. coli* primers, 100 nM of ITS3 and ITS4 primers, or 300 nM of HCMV primers), and a serial dilution (10^4^–10^0^ copies/reaction) of gDNA of *E. coli, S. cerevisiae*, or HCMV at 95 °C for 3 min, cycled at 95 °C for 30 s, 60 °C for 30 s, and 72 °C for 30 s for 50 times. All the PCR assays in this work followed the same optimal conditions unless otherwise specified.

#### Investigation of specificity and multiplexity of multiplex PCR assay

Duplex or triplex real-time PCR assays in 1× PCR SYBR Green master mix were processed. In duplex real-time PCR assays, two pairs of primers and two corresponding templates of gDNA (combinations of each two gDNA: 10^6^ copies of *E. coli*, 10^6^ copies of HCMV, and 10^5^ copies of *S. cerevisiae*) were included in a 25 µL of final PCR mixture. In a triplex real-time PCR assay, three pairs of primers and three corresponding templates of gDNA (10^6^ copies of *E. coli*, 10^6^ copies of HCMV, and 10^5^ copies of *S. cerevisiae*) were included in a 25 µL of PCR mixture. Melting curves were used to identify the specificity and multiplexity of the multiplex PCR assay, which were generated at an increase in temperature from 72 to 95 °C using 0.1 °C increments for 5 s between each step after the final elongation of amplification reaction.

#### Amplicon detection

Five hundred nanograms of biotinylated double-stranded target DNA interacted with 10 µL of brown streptavidin magnetic beads for 5 min at room temperature, then were denatured and purified to collect the single-stranded target DNA bound with beads. Once the filter paper printed with probes was dipped into the detection solution, the target DNA with beads flowed through the white paper via capillary force, hybridized the complementary probe sequence, and formed a specific brown signal. The final volume of detection was 100 µL in 1× PBS-T. All the detection work in this study followed the same volume.

#### Investigation of probe sequence length

The printed probe sequence was structured as a segment of pathogen gDNA sequence with fifteen thymine bases and amino-modifier C12 at 5′ end (Table S2). Three microliters of each probe were printed on the activated filter paper manually. After incubation, blocking, and washing, detection was completed on each printed paper using 500 ng of *S. cerevisiae* amplicons bound with 10 µL of magnetic beads. The signal difference between the intensity captured by each length (35, 45, 55 or 65 bases) of *S. cerevisiae* printed probe (Table S3) and the intensity captured by NTC on the same paper slide was investigated and compared.

#### Singleplex, mixed, and multiplex amplicon detection

To prepare the spiked DNA (sDNA), 10^6^ copies of each gDNA was added into 200 µL of human serum, and sDNA was extracted using the Qiagen Blood DNA Mini kit following the manufacturer’s instructions. Both singleplex and multiplex PCR assays in 1× Kapa PCR readymix were used to prepare the detection targets on a Techne primer thermal cycler. The detection with the mixed targets was processed by mixing two or three of singleplex PCR products. The multiplex detection was processed with the PCR products from duplex or triplex PCR assay. Five hundred nanograms of target DNA bound with 10 µL of magnetic beads was used for each detection. The average intensity of each specific signal was compared to that of NTC on the same paper. Each set of conditions was completed in triplicate.

#### Limit of detection based on singleplex, duplex, and triplex amplicons

Ten nanograms, 50 ng, 100 ng, or 500 ng of either a single target (F, V, or B) or a multiplex target (FV, VB, FB, or FVB) bound with 10 µL of magnetic beads were used to hybridize with either the specific single probe or multiple probes printed on the functionalized filter paper. The intensity difference between each specific and NTC signal on the same paper slide was compared to confirm the quality range of the detection limit analysis. The signal intensities detected using 10 ng and 50 ng of amplicons were further measured in triplicate. The limit of detection in this work was confirmed by comparing the average intensity of each specific signal to that of NTC on the same paper.

#### Imaging and statistical analysis

The signal intensity of each printing location was captured using MyImageAnalysis software. Graphs were generated using Excel and CorelDRAW^23,28^. Statistical significance of more than two intensities in one group was analyzed using one-factor Analysis of Variance (ANOVA) at significance level of 0.05 and Bonferroni Correction was used to complete multiple comparison analysis. Statistical significance comparing two intensities in a group was analyzed using student’s t-test, where applicable (*p* ≤ 0.05).

## Results and discussion

In this study, we optimized the length of probe immobilized on the filter paper surface; designed specific primers of one bacterium (*E. coli*), one fungus (*S. cerevisiae*), and one virus (HCMV); and confirmed the corresponding optimal concentration of each pair of primers and the universal annealing temperature in one multiplex PCR system. The functionalized filter paper can simultaneously detect the three amplified products from different species of DNA (*E. coli*, *S. cerevisiae*, and HCMV) in one test, which are the common pathogens of polymicrobial BSI in patients with neutropenia^29–36^.

### Investigation of printed probe length

**Figure 1:**
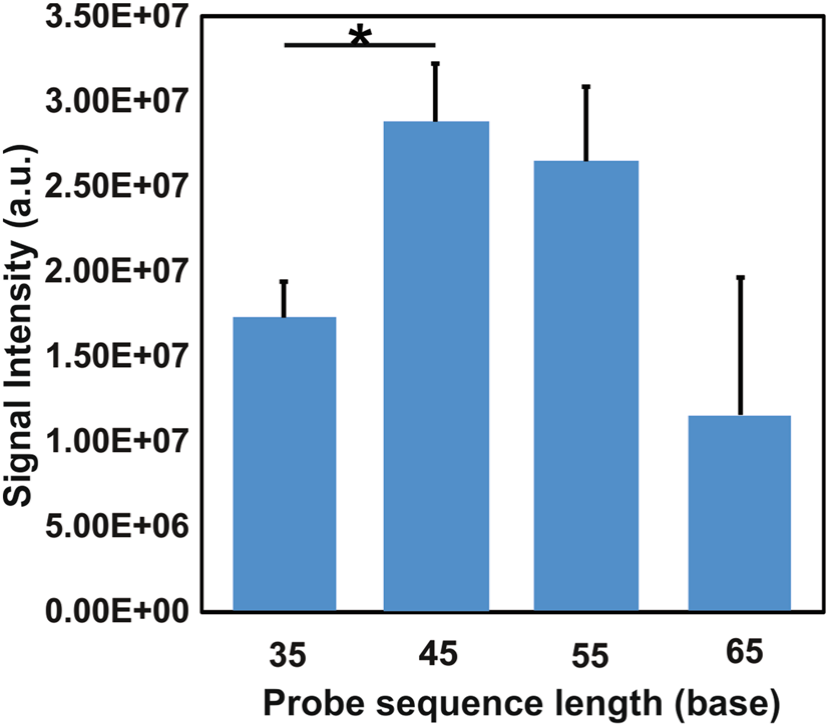
Detection effect comparison based on the length of printed probe sequence. The strongest signal was captured by the 45-base probe. *p ≤ 0.05/comparison times (post hoc test).

The goal of printing a probe on the filter paper is to capture a specific complementary target in detective solution and hybridize with it to form a double helix. Stability and specificity of DNA hybridization are impacted by several factors, such as hybridization temperature, time, platform, complexity of the probe or target, washing solution composition, washing conditions, among others^37,38^. The hybridization in our method occurred at room temperature, on the surface of a cellulose filter paper, and in a short period of time. We have previously optimized washing solution and conditions^23^, and investigated the hybridization specificity based on the different mutation rates of a target sequence^28^. In this study, we compared the hybridization effect based on four different lengths of *S. cerevisiae* specific probe sequence. All detection showed target-specific visual signals. Both visual detection and quantity analysis based on the 45-base probe displayed the strongest signal intensity (Fig. 1). ANOVA revealed a significant difference in the average intensities between these four groups (*p* < 0.05). Post-hoc analysis revealed that the signal intensity captured by the 45-base probe was significantly stronger than that of the 35-based probe. One of the most likely reasons would be that the length of 45 bases helps the cooperative transient hybridization on the functionalized filter paper surface to form the most stable conformation. However, the complexity of longer probes may challenge this cooperativity by increasing thermodynamic driving forces of hybridization at room temperature, for example, by extending the formation time of the correct initial base-pair and complicating the following events of stacking and base pairing influenced by neighboring pairs^39,40^. Considering hybridization efficiency in the given condition and cost of detection, we selected the length of 45 bases to design all the probes in the downstream work. To avoid background noise resulting from primer dimers, all the probes for subsequent experiments were designed to differ from primer sequences.

### Optimization of PCR assay and construction of standard curve

**Figure 2:**
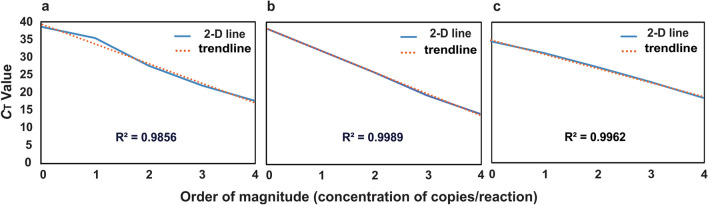
Standard curves of three qPCRs (Target: (**a**) *Escherichia coli*, (**b**) *Saccharomyces cerevisiae*, and (**c**) Human cytomegalovirus; Primer: (**a**) specific primers of *Escherichia coli*, (**b**) ITS3/4 fungal primers, and (**c**) specific primers of Human cytomegalovirus) display negative linear relationships between the initiated copies of each pathogen and the corresponding C_*T*_ values. R^2^ values indicate the sufficient quality of primers to yield the reliable experimental data.

Blood culture is suboptimal to identify polymicrobial BSI for various reasons, such as cultivability of pathogens, the low quantity of pathogens, or the different stages of infection^41,42^. To simultaneously identify multiple pathogens from one sample in one test, we developed a multiple detection model using activated cellulose filter paper. *E. coli*, *S. cerevisiae*, and HCMV are pathogenic microorganisms commonly occurring in the neutropenic patients with BSI and are associated with a significant increase in mortality rate^29,31,34^. In our amplification system, the optimal concentrations of these three pairs of primers were 200 nM (*E. coli*), 100 nM (*S. cerevisiae*), and 300 nM (HCMV). The universal annealing temperature of these three pairs of primers was 60 °C. All three standard curves based on a tenfold serial dilution of gDNA showed negative linear correlations between the copies of initiated DNA and cycle threshold (*C*_*T*_) (Fig. 2). R squared values for *E. coli*, *S. cerevisiae*, and HCMV were calculated as 0.98, 0.99, and 0.99, respectively, to verify the reliability of the reactions. We also investigated accuracy of multiplex real-time PCR assays based on these three gDNA. Both duplex and triplex real-time PCR assays showed target-specific amplification based on melting curve analysis (Fig. S1).

### Investigation of singleplex, mixed, and multiplex amplicon detection

**Figure 3:**
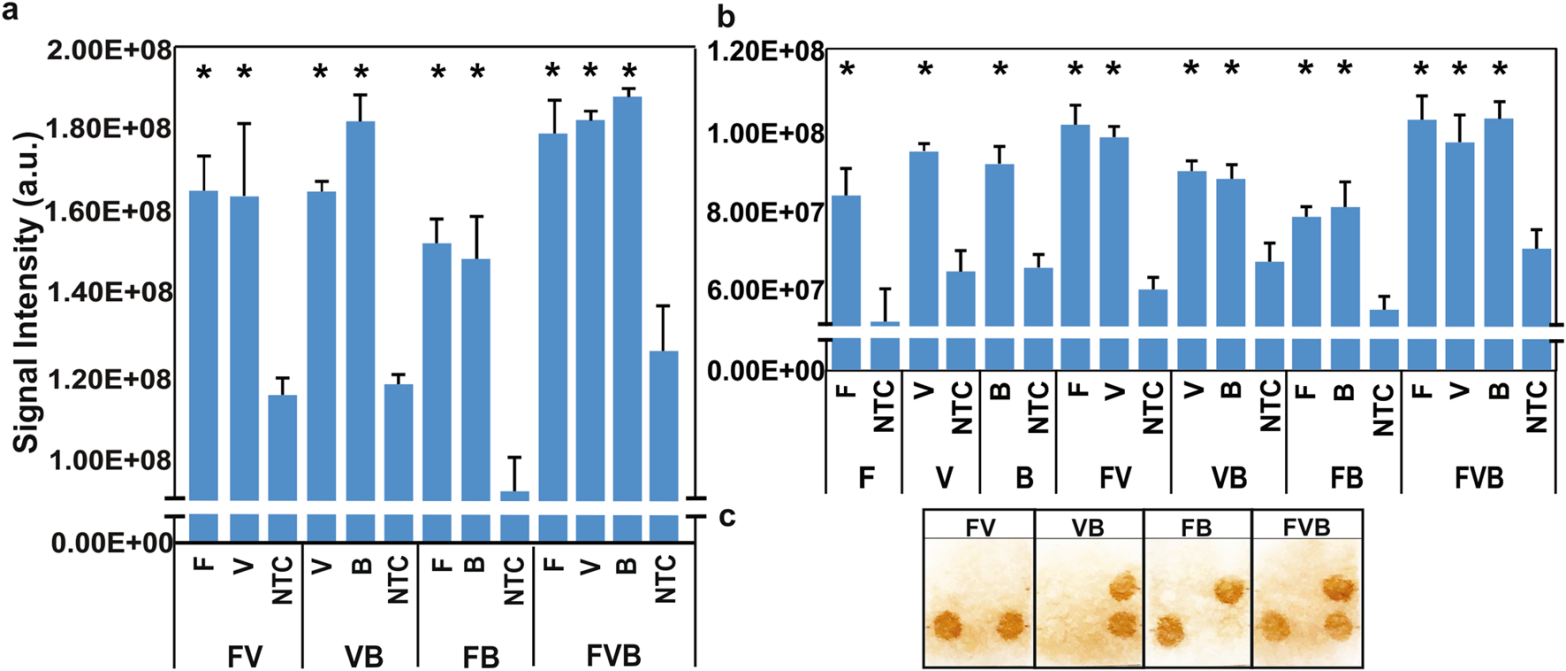
Detection with different types of targets. (**a**) Signal intensities of multiplex PCR products (FV, VB, FB, and FVB) amplified from genomic DNA. (**b**) Signal intensities from a single PCR product (F, V, and B) to a mixture of two or three single PCR products (FV, VB, FB, or FVB) amplified from spiked DNA. (**c**) Visual detection using multiplex PCR products amplified from spiked DNA. All the differences of intensities between the specific and the NTC signals on the same filter paper were significant. X axes of 3a and 3b represent the targets of detection. Y axes display the signal intensities. F: *Saccharomyces cerevisiae*. V: human cytomegalovirus. B: *Escherichia coli*. NTC: negative control. *p ≤ 0.05 using student t-test (F, V, and B) or *p* ≤ 0.05/comparison times using post hoc test (FV, VB, FB, or FVB).

PCR products amplified either from gDNA or sDNA of *E. coli* (B), *S. cerevisiae* (F), and HCMV (V) were detected using the corresponding probe (Table S2) printed on filter paper. The target was 500 ng of each singleplex PCR product (F, B, or V), or of the mixture of singleplex PCR products (FV, VB, FB, or FVB), or of the multiple targets from multiplex PCR (FV, VB, FB, or FVB). All the amplicon detection from both gDNA (Fig. 3a) and sDNA (Fig. 3b,c) displayed clear and specific signals from a singleplex target to a mixed target, or to a multiplex target. Figure 3 shows that the difference of intensity between each target signal and the negative control of APT_2_ on the same paper is significant. All the specific single or multiple target signals were possible to be identified on site by the naked eye as shown in Fig. 3c.

### Evaluation of limit of detection

**Figure 4:**
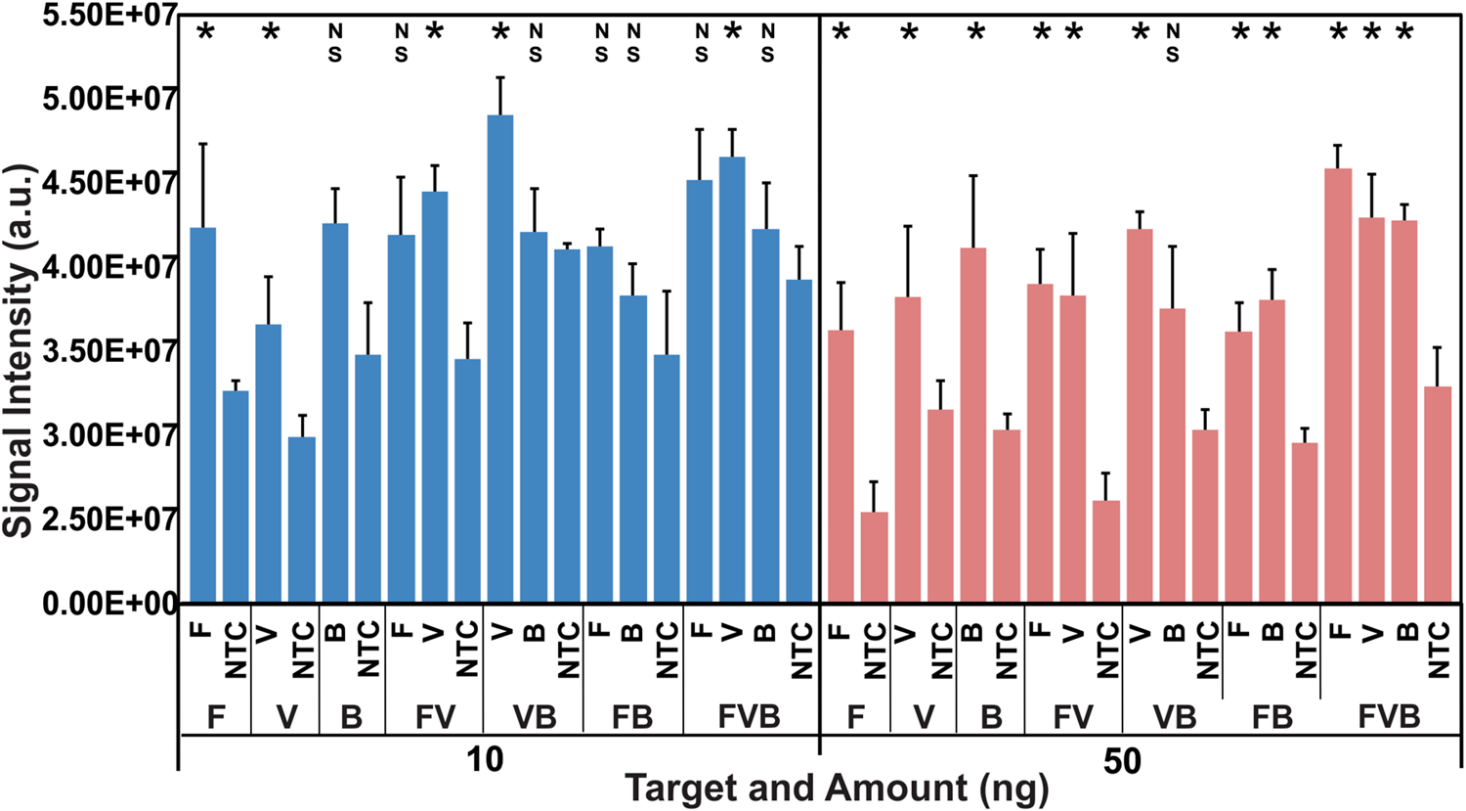
Detection limit (DL) analysis of the functionalized filter paper based on PCR products amplified from genomic DNA. F, V, or B represents a single PCR product. FV, VB, or FB is the corresponding duplex PCR product. FVB means the triplex PCR product. The signal intensities of blue columns represent detection outcome when 10 ng of each target was used, and the pink ones represent the results when 50 ng of each target was used. Every signal intensity was compared to that of NTC on the same paper slide. The work was done in triplicate. The DL was between 0.1 ng/µL and 0.5 ng/µL. F: *Saccharomyces cerevisiae*. V: human cytomegalovirus. B: *Escherichia coli*. NTC: negative control. *p ≤ 0.05 using student t-test (F, V, and B) or *p* ≤ 0.05/comparison times using post hoc test (FV, VB, FB, or FVB). NS: no significant difference.

PCR products amplified from gDNA were used to estimate the limit of detection using the activated filter paper tool (Figs. 4 and S2). Seven printed filter paper slides, three different singleplex amplicons (F, V, B), and four different multiplex amplicons (FV, VB, FB, and FVB) were used to complete the detection. The detection signals of four target quantities—10 ng, 50 ng, 100 ng, and 500 ng—were compared. Increasing signal intensity was displayed as the increase in target quantity (Fig. S2). For a singleplex PCR product detection, by comparing the average specific intensity to that of NTC on the same paper, 2 (F and V) of 3 (F, V, and B) comparisons showed statistically significant differences (*p* < 0.05) when 10 ng of each target was used; however, all three differences were significant when 50 ng of each target was used (*p* < 0.05). For a multiplex PCR product detection, by comparing the average specific intensity to that of NTC on the same paper, only three (FV_V, VB_V, and FVB_V) differences were statistically significant after post hoc analysis when 10 ng of each multiplex amplicon was used, while all the differences were statistically significant only except that of VB_B when 50 ng of each multiplex target was used (Fig. 4). The current detection limit of this tool was between 0.1 ng/µL (10 ng of each target) and 0.5 ng/µL (50 ng of each target) based on the manual probe printing, which may be further improved when robotic printing is used^43^.

Rapid and accurate pathogen diagnosis is crucial in initiation of appropriate antimicrobial treatment. The main advantage of NAAT-based diagnosis of BSI is to reduce turnround time and to increase the sensitivity and specificity of pathogen diagnosis even after antibiotic treatment to guide the early BSI regimens^20^. To develop a rapid, portable, and cost-efficient pathogen detection tool, NAATs can be combined with some cost-efficient materials such as paper^23,44–46^. Cellulose filter paper composed of cellulose fibers possesses a three-dimension porous structure and can be functionalized with chemicals, which enable a stable immobilization of a higher concentration of probe DNA, a higher flux of targets for pathogen detection, and an automatic detection via capillary force created by the paper micropores when compared to the glass surfaces^47^. Cellulose filter paper also has more options for pore size than nitrocellulose, making it suitable for molecular target binding 1 µm magnetic beads in diameter. Moreover, the high porosity and multiple pore size options of the cellulose filter paper enable it to produce rapid multiplex detection through instantaneous wicking force^46,48^.

NAAT techniques are more sensitive than blood culture, however, diagnosis provided by NAATs must exclude any possible contamination from degraded or inactivated pathogens^20^. This suggests that, without increasing the cost, the current NAAT-based and blood culture-based diagnostic methods of BSI are mutually complementary, particularly for polymicrobial BSI. An ideal BSI regimen might be based on rapid results from NAATs and physicians’ clinical evaluation, and can be further optimized by integrating the results from blood culture^20^.

We have verified that the developed diagnostic tool using cellulose filter paper is suitable for rapid identification of multiple pathogens’ DNA simultaneously. The tool is cost-efficient (approximately $3 per triplex assay), easy to manufacture, easy to carry, easy to operate, and biodegradable^23,28^. All the detection steps (from printing to detection) included in this method were operated manually and have potential to be integrated into an automatic sample-to-result portable equipment for polymicrobial BSI diagnostics. Emerging technologies in point-of-care biosensing systems such as described here and by other groups^49–51^ have potential application in point-of-care medical diagnosis, which is in huge demand^52–55^.

In the future, this work can also be expanded to different microorganisms and their antimicrobial susceptibility testing. Utilizing isothermal amplification in this system would allow instrument-free detection.

## Conclusions

Initiating appropriate antimicrobial treatment within 1–3 hours from BSI symptom onset is crucial to save the patients’ lives; however, it is a big challenge only relying on the current routine diagnostic method—blood culture. We demonstrate the utilization of cellulose filter paper to develop a rapid pathogen DNA detection tool based on NAATs, which is able to identify multiple species of pathogen DNA simultaneously on site in 1–5 min. The developed tool could be integrated into a sample-to-result device to complete pathogen diagnostics, which would facilitate the diagnosis of polymicrobial BSI and support clinicians’ decisions in selecting targeted antimicrobial therapy.

## Supplementary Information

Supplementary Information.

## Data Availability

Data presented in this paper will be made available on request subject to a formal data sharing agreement.
