# Supplementary material for: Rapid DNA visual detection of polymicrobial bloodstream infection using filter paper
Source: Sci Rep. 2022 Mar 16;12:4515. doi: 10.1038/s41598-022-08487-4 (PMC8927095; doi:10.1038/s41598-022-08487-4)
Supplement: Supplementary file 1 — Supplementary Information. [file 41598_2022_8487_MOESM1_ESM.pdf]

SUPPLEMENTARY MATERIAL

Rapid DNA visual detection of polymicrobial bloodstream infection using filter paper

Yajing Song[1*], Peter Gyarmati[1]

[1] Department of Cancer Biology and Pharmacology, University of Illinois College of Medicine, Peoria, IL, USA.

[*] Correspondence: yajings@uic.edu

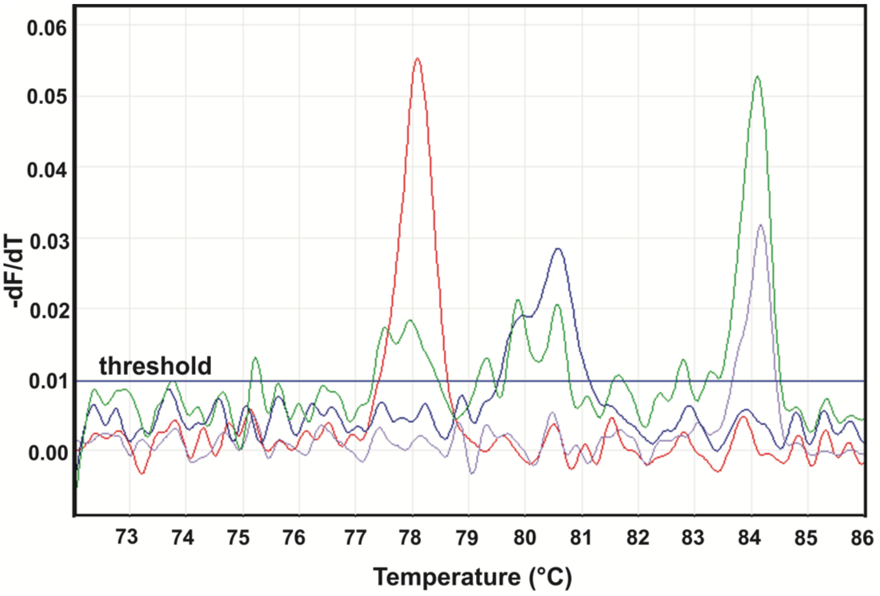

Fig. S1 Melting curve analysis in a triplex PCR assay based on SYBR Green. Compared to the reference melting curves of *Escherichia coli* (red), *Saccharomyces cerevisiae* (blue), and Human cytomegalovirus (purple), the triplex real-time PCR melting curve (green) displays the three specific melting peaks. dF/dT: negative derivative of fluorescence with respect to temperature

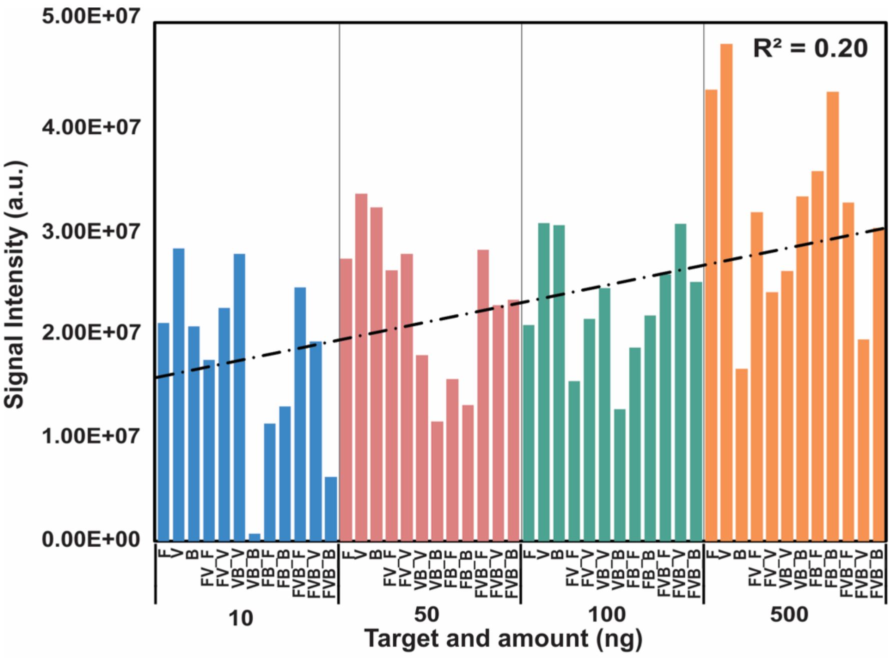

Fig. S2 Detection effect comparison of different amounts of singleplex (F, V, and B), duplex (FV, VB, and FB), and triplex (FVB) amplicons. As described in the main text, each signal intensity was calculated by subtracting the negative control intensity from that of the specific target on the same filter paper. The detection effect of four quantities (10 ng_blue, 50 ng_pink, 100 ng_green, and 500 ng_yellow) was compared. Seven amplicons and twelve signals were included in each quantity group. The signal intensity shows an increasing trend as the increase in target quantity ($R^2 = 0.2$). F: *Saccharomyces cerevisiae*, V: human cytomegalovirus, B: *Escherichia coli*

Table S1 The primer sequences and modifications of *Escherichia coli*, *Saccharomyces cerevisiae*, and Human cytomegalovirus

		**5' terminal modification**	**Sequences**
Escherichia coli	Forward	Biotin	CCCCGACAGGGTGAAAACTA
	Reverse	None	GGTGCCAACCGAATTTCCTG
Saccharomyces cerevisiae	Forward	Biotin	GCATCGATGAAGAACGCAGC [27]
	Reverse	None	TCCTCCGCTTATTGATATGC [27]
**Human cytomegalovirus**	Forward	Biotin	GCTACAATAGCCTCTTCCTCATCTG [26]
	Reverse	None	CATGTACGGGGGCATCTCTC

Table S2 The sequences and their modifications of printed probes (*Escherichia coli*, *Saccharomyces cerevisiae*, and Human cytomegalovirus)

	5' terminal modification	Carbon spacer	Polythymine spacer	Main sequences
Escherichia coli	Amino group	C12	PolyT (15)	CGATTTCACTTATCCCTTTCCCTCTTGCAC
Saccharomyces cerevisiae	Amino group	C12	PolyT (15)	GAGGTCAAACTTTAAGAACATTGTTCGCCT
**Human cytomegalovirus**	Amino group	C12	PolyT (15)	AGCCTGAGGTTATCAGTGTAATGAAGCGCC

Table S3 The probe sequences and modifications of *Saccharomyces cerevisiae* for length optimization

Length	5' terminal modification	Carbon spacer	Polythymine spacer	Main sequences
**35-base**	Amino group	C12	PolyT (15)	TCCTCCGCTTATTGATATGC
**45-base**	Amino group	C12	PolyT (15)	TCCTCCGCTTATTGATATGCTTAAGTTCAG
**55-base**	Amino group	C12	PolyT (15)	TCCTCCGCTTATTGATATGCTTAAGTTCAGCGGGTACTCC
**65-base**	Amino group	C12	PolyT (15)	TCCTCCGCTTATTGATATGCTTAAGTTCAGCGGGTACTCCTACCTGATTT
